# Efficacy, Safety, and Retention Rate of Extended-Release Divalproex Versus Conventional Delayed-Release Divalproex: A Meta-Analysis of Controlled Clinical Trials

**DOI:** 10.3389/fphar.2022.811017

**Published:** 2022-04-05

**Authors:** Chen Qi Zhang, Hong Yan Li, Yong Wan, Xue Yang Bai, Lu Gan, Juan Wang, Hong Bin Sun

**Affiliations:** ^1^ Department of Special-Need Medical, Chengdu BOE Hospital, Chengdu, China; ^2^ Department of Neurology, Sichuan Academy of Medical Sciences and Sichuan Provincial People's Hospital, Chengdu, China

**Keywords:** divalproex, extended release, efficacy, safety, meta-analysis

## Abstract

**Purpose:** A novel once-daily divalproex-extended release (ER) dose formulation has been developed; this formulation prolongs the therapeutic serum levels of the drug, compared with the twice-daily conventional divalproex-delayed release (DR) formulation. This study aimed to systematically examine and compare the efficacy, safety, and retention rates of the ER divalproex (VPA-ER) and conventional DR divalproex (VPA-DR) formulations.

**Methods:** Randomized control trials (RCTs) reporting the efficacy, adverse events (AEs), and medication compliance of ER and DR divalproex were searched in online databases, including PubMed, Embase, and Cochrane Library databases, by searching MeSH words and term words. Observational studies with potential biases were excluded. The meta-analysis was performed using Stata 16.0 software.

**Findings:** Thirteen RCTs, involving 1,028 participants, were included in this meta-analysis. Efficacy, AEs, and drug retention rates were the main study outcomes. According to our study, VPA-ER presented clinically significant benefits compared with the placebo in the population with bipolar disorder (BD) (39.5% *versus* 27.2%, *p* < 0.001). A similar efficacy of VPA-ER and VPA-DR in controlling seizures was observed in epilepsy patients (87.4% *versus* 86.5%, *p* = 0.769). A significantly lower incidence of AEs was reported in the VPA-ER group than in the placebo group (26.8% *versus* 34.8%, *p* = 0.003). By contrast, there was no evidence of difference in safety between VPA-ER and VPA-DR (29.4% *versus* 30.5%, *p* = 0.750). In addition, the drug retention rate was significantly lower in the VPA-ER group than in the placebo group (76.0% *versus* 82.7%, *p* = 0.020), especially in migraine patients (*p* = 0.022) and in patients who were treated for fewer than 4 weeks (*p* = 0.018).

**Implications:** The efficacy of VPA-ER was significantly superior to that of the placebo treatment, which provided efficacy similar to that of conventional VPA-DR. VPA-ER is well tolerated with a low rate of AEs compared to the placebo. In addition, the acceptable medicine compliance of VPA-ER was conducive to the long-term maintenance treatment of chronic diseases. Although we analyzed open labels and crossover design RCTs, large-scale multicenter studies on the efficacy and medicine compliance of new ER formulations with less AEs are required to validate our conclusion.

## 1 Introduction

Valproic acid and its derivatives, including valproate sodium (sodium valproate), divalproex, and divalproex sodium, are all known as valproate (VPA). VPA is a weak sodium channel blocker that produces weak inhibitors of enzymes to inactivate gamma-aminobutyric acid (GABA)-like aminobutyric aminotransferase (Cipriani et al., 2013). In addition, VPA can regulate serotonergic and glutamatergic transmission, energy metabolism, neuronal membrane lipid synthesis, and neurotrophic and neuroplastic effects (Winterer and Hermann, 2000; Schloesser et al., 2008).

VPA is the most commonly applied first-generation broad-spectrum antiepileptic drug (AED) used to treat generalized and focal epilepsies in children and adults and has been approved by the Food and Drug Administration (FDA). Divalproex has been approved for use in the United States since 1983 (Sommerville et al., 2003). It has a broad-spectrum medicine used in the treatment of bipolar depression and rapid cycling, psychotic symptoms, impulsive aggression, post-traumatic stress disorder (PTSD), neuropathic pain, and the prophylaxis of migraine headaches (Ross, 2000; Davis et al., 2004; Adamou et al., 2007; Bialer and Yagen, 2007; Soares-Weiser et al., 2007; Wang et al., 2016). Epilepsy, bipolar disorder (BD), and migraine are three common chronic diseases that require long-term therapy, depending on the optimal regimen, dose, and patient compliance (Genton, 2005). Many AEDs, including VPA, have short half-lives and must be administered several times daily, with large fluctuations in peak-to-trough plasma concentrations (Dutta and Zhang, 2004), resulting in poor pharmacokinetic (PK) properties, adverse events (AEs), and unsatisfactory adherence (Leppik and Hovinga, 2013).

VPA has been approved in several formulations, including the original delayed-release tablet (e.g., Depakote), enteric-coated particles, sprinkle capsules, sustained-release tablets (e.g., Depakine Chrono), and a more recently approved extended-release tablet (e.g., Depakote ER). In the present study, VPA-ER and VPA-DR were defined as extended-release and delayed-release formulations of VPA, respectively. In ER formulations, the dosing interval is usually extended to minimize the dosing frequency (Leppik and Hovinga, 2013). In addition, they can potentially minimize spikes in the maximum plasma concentrations (C_max_) at a steady state and maintain a relatively constant plasma drug concentration. Moreover, they can minimize concentration-related AEs (Bialer, 2007). Once-daily VPA-ER is characterized by a hydrophilic polymer matrix controlled-release tablet system that allows the slow release of drugs in the stomach, small intestine, and large intestine for 18–24 h (Brandt and May, 2018). Compared with the standard twice-daily DR formulation, once-daily VPA-ER significantly stabilizes serum levels without marked peak-to-trough fluctuations and reduces the dosing frequency and the possibility of dosing flexibility, which improves patient compliance, satisfaction, and ultimately the quality of life (Davis et al., 2004; Genton, 2005).

Although once-daily formulations are more convenient than multiple doses per day, potential subtherapeutic concentrations following delayed or missed doses should be considered. Once-daily formulations are unable to improve therapeutic coverage because they cannot pharmacokinetically maintain the effective drug concentration in biological fluids and tissues. A missed dose of once-daily formulation usually has a greater influence on treatment because of the higher dose than that of the multiple-dose formulation. Therefore, the risk of breakthrough seizure is higher in once-daily AED administration than that in twice-daily administration (Dutta and Zhang, 2004; Ahmad et al., 2005). VPA-ER is limited by a high daily dose owing to its low bioavailability. A mean increase in the daily dose of divalproex ER at 12% (8–20%) can achieve an equivalent plasma exposure level to that of divalproex DR (Dutta and Zhang, 2004; Dutta et al., 2006).

Currently, ER formulations are preferred for the treatment of EP and prevention of migraine owing to better compliance, convenience, and consistent plasma concentration over time. To our knowledge, comparative conversion studies of VPA-ER and conventional VPA-DR are lacking. Small-sample studies analyzing the safety and efficacy of VPA-ER in different populations remain controversial. Therefore, this study aimed to investigate the efficacy, safety, and retention rate of VPA-ER and VPA-DR using meta-analysis.

## 2 Methods

This study did not require ethical approval since no subjects were recruited. According to PRISMA (Liberati et al., 2009) principles and MOOSE (Stroup et al., 2000) guidelines, the search strategy, selection criteria, data extraction, quality assessment, and statistical analysis were predesigned based on Cochrane Review Methods. We registered this study using INPLASY 2021110090, and the DOI number is 10.37766/inplasy 2021.11.0090 (https://inplasy.com/inplasy-2021-11-0090/).

### 2.1 Searching Strategy

Two researchers were independently responsible for searching eligible RCTs in online databases, including PubMed (published from 1983 to October 2021), Embase (published from 1982 to October 2021), and Cochrane Library (published from 2001 to October 2021) without language limitations. Relevant RCTs were searched through a combination of MeSH words and term words, including “Valproic acid”[MeSH], “divalpro*,” “Valpro*,” “Depak?e,” “Semisodium Valproate,” “Modified-release,” “Delayed-release,” “Extended-release,” “sustained-release,” “prolonged-release,” “controlled-release,” “treatment [MeSH],” “Therapeutics [Mesh],” “therap*,” and “Treatment*” (Supplementary Materials—additional file for full electronic search). References in each eligible study were also manually reviewed to avoid missing data.

### 2.2 Inclusion and Exclusion Criteria

Inclusion criteria were given as follows: 1) randomized controlled blind/double-blind, crossover, open-label trials reporting the efficacy, safety, and medication compliance (e.g., reduction in epileptic seizure frequency) of VPA-ER and the control were included. 2) Patients receiving VPA-ER without disease limitation.

The exclusion criteria were as follows: 1) observational studies, cohort studies, reviews, case reports, letters, communication, editorials, lectures, and conference abstracts; 2) unclearly defined divalproex ER; and 3) studies with missing or repeated data.

### 2.3 Data Extraction

Two independent investigators (ZCQ and YW) individually screened the literature and extracted and evaluated the data. Any disagreement was resolved by discussion with a third reviewer (LHY or SHB). Planned data extraction included the following items: authors, year of publication, study design, study population (e.g., age, sex, sample size, and disease type), intervention measures (e.g., type of VPA, dose, mode of administration, and duration), comparison details, clinical outcome indicators (e.g., efficacy and retention rate), AEs, study methodology (e.g., method of randomization and blinding), and other characteristics.

### 2.4 Quality Assessment

Two reviewers (ZCQ and HYL) independently reviewed the quality of the included literature using Cochrane Handbooks (http://community.cochrane.org/handbook), including the following seven aspects: random sequence generation, allocation concealment, blinding of participants and personnel, blinding of assessors, incomplete outcome data, and selective outcome reporting.

### 2.5 Statistical Analysis

Stata 16.0 software (STATA Corporation, College Station, TX) was used to perform the statistical analyses. Dichotomous variables were analyzed using the Mantel–Haenszel method and were expressed as risk ratios (RRs) with 95% confidence intervals (CIs), which examined the effectiveness, incidence of AEs, and drug retention rate between different groups.

The Q test and chi-square test were used to estimate statistical heterogeneity using the *p* value and I^2^ statistic. *p* < 0.05 and I^2^ > 50% were the criteria used to classify the data as heterogeneous, and data were analyzed using a random-effects model; otherwise, we used a fixed-effects model in the meta-analysis (*p* > 0.05, I^2^ ≤ 50%). Statistical significance was defined as a two-tailed *p*-value < 0.05.

For subgroup analysis and investigation of heterogeneity, we performed the following predefined subgroup analyses if sufficient data were available: different types of diseases, periods of treatment, and other outcome measures.

Had sufficient data been available, we would have examined the robustness of the meta-analysis by conducting a sensitivity analysis. Finally, both Begg and Egger tests were performed to identify publication bias.

## 3 Results

### 3.1 Included Studies

**FIGURE 1:**
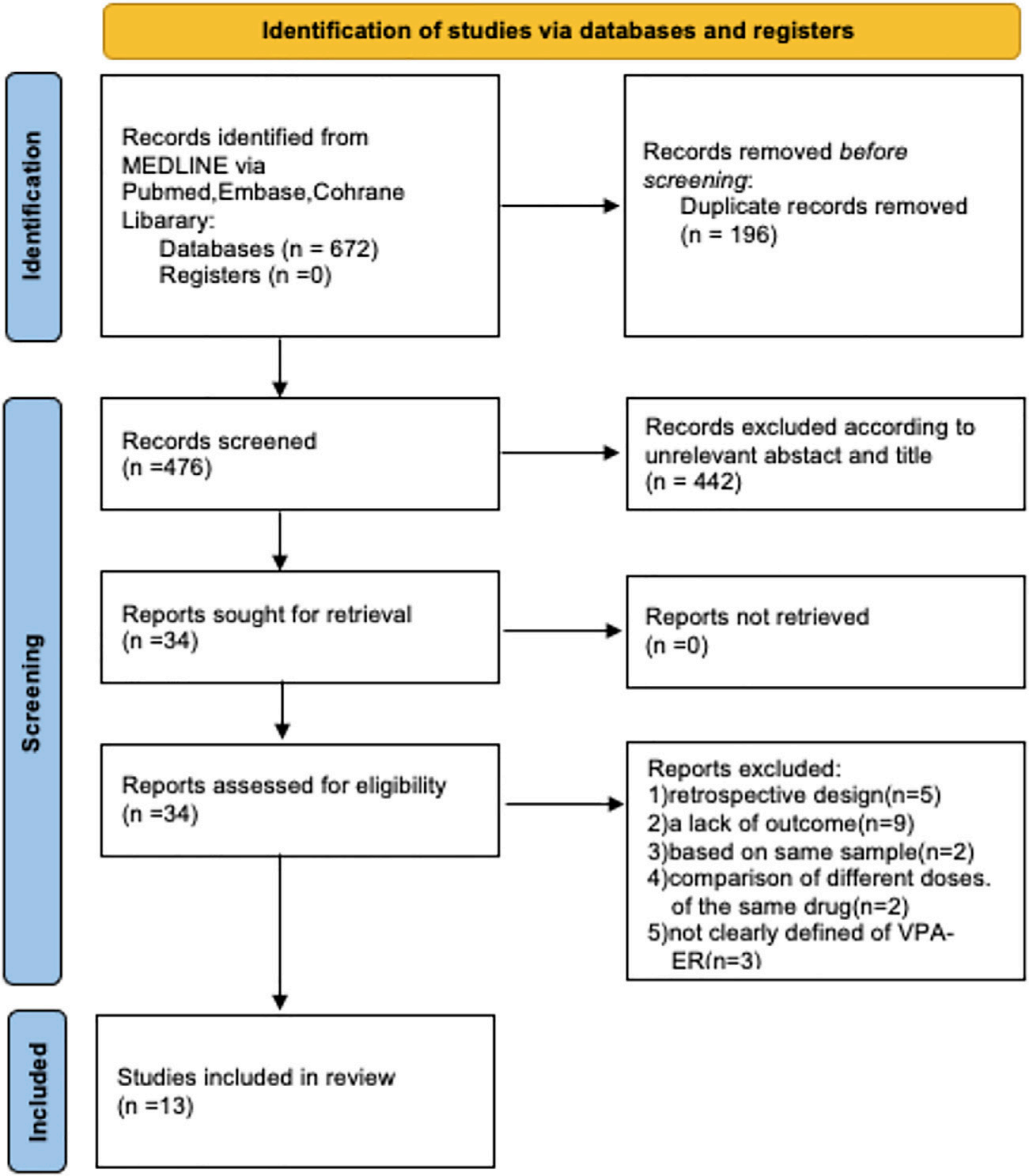
Flowchart of selection studies for this meta-analysis.

A total of 672 records were initially obtained following an online search; after excluding duplications, 476 remained (Figure 1). We excluded non-RCTs and identified 34 human clinical trials. Following a review of the full text, a total of 13 eligible RCTs, involving 1,028 subjects, were finally selected for the following meta-analysis.

Notably, one study (Apostol et al., 2008) had a parallel design with three separate studies with different doses of the same drug (Depakote ER: 250 mg/d, 500 mg/d, 1,000 mg/d), compared with the placebo, to examine the effects of dose on clinical efficacy and safety. Consequently, we defined them as three trials. In addition, there were three types of administration schedules employed in one study (Hirschfeld et al., 2010): ChVbid, ChVom, and ChVoe, given the influence of these modes on AEs and efficacy. Consequently, we also considered this study as three trials. At last, 17 studies were recruited in the present study.

A total of 1,028 patients receiving VPA-ER were analyzed, including EP patients in four RCTs (Thibault et al., 2002; Sommerville et al., 2003; Kernitsky et al., 2005; Herranz et al., 2006), BD patients in six (Bowden et al., 2006; Ghaemi et al., 2007; Wagner et al., 2009; Hirschfeld et al., 2010; McElroy et al., 2010; Muzina et al., 2011), and MA patients in three (Jensen et al., 1994; Freitag et al., 2002; Apostol et al., 2008). Most RCTs were multicenter, and patients were mainly from the United States. The sample size of each trial ranged from 16 to 377, with ages ranging from 5 to 73 years, and that of VPA-ER-treated patients with a mean dose ranging from to 250–2,961 mg/day. The treatment period ranged from 11 days to 16 months (Table 1).

**TABLE 1:** Basic Characteristics of all included studies.

Study ID	Study design	No. randomed (→analyzed)	Mean age (year)	Gender (F:M)	Population (Diagnosis)	(A) Treatment intervention + mean dose	Duration time	Efficacy (%)	Retention rate (%)	AE (%)
						(B) Control intervention + mean dose				
Apostol et al., 2008	RCT/placebo controlled	304(82/74/75/73)	(A1) 14.2 ± 1.69	(A1) 52:29	—MA(IHS)	(A1) Depakote ER, 250 mg/d, once daily	4 w	—migraine HA rate (Subjects with at least 50% reduction) (A1): 33(41%)	(A1) 74 (90%)	(A1):53(65%)
			(A2) 14.2 ± 1.56	(A2) 40:34		(A2) Depakote ER, 500 mg/d, once daily		(A2): 27 (36%)	(A2) 62 (84%)	(A2):53(72%)
			(A3) 14.3 ± 1.66	(A3) 34:39		(A3) Depakote ER, 1,000 mg/d, once daily		(A3): 37 (51%)	(A3) 62 (83%)	(A3):48(64%)
		299(81/74/73/71)	(B) 14.2 ± 1.50	(B) 37:34		(B) placebo		(B): 33 (46%)	(B) 67 (92%)	(B):42(58%)
Bowden et al., 2006	RCT/placebo controlled	377(185/192)	(A) 37.0 ± 10.71	(A) 74:113	—BD(DSM-IV-TR)	(A) divalproex ER 3,057 mg/d (95.9 μg/ml)	3 w	—MRS (at least 50% improvement from baseline in MRS score): (A): 90(48%)	(A) 173 (90%)	(A):162(84%)
		364(177/187)	(B) 38.1 ± 10.28	(B) 81:96	—MRS: (A) 26.6 ± 5.6 (B) 26.6 ± 5.6	(B) placebo		(B):60( 34%)	(B) 179 (96.8%)	(B):134(72%)
Freitag et al., 2002	RCT/placebo controlled	237(122/115)	(A) 39.8 ± 11.24	(A) 97:25	—MA(IHS)	(A) Depakote ER, 871 mg/d	17 w	M—migraine HA rat Subjects with at least 50% duction): (A): 36(30%)	(A) 101(82.8%)	(A):83(68%)
		202(101/101)	(B) 41.3 ± 11.97	(B) 90:25	—Baseline phase 4-wk migraine headache rate: (A) 4.4 ± 1.62 (B) 4.2 ± 1.94	(B) placebo		(B): 28(24%)	(B) 101(87.8%)	(B):81(70%)
Ghaemi et al., 2007	RCT/placebo controlled	20(11/9)	(A)32.7 ± 2.3	(A)7:3	—BD(DSM-IV)	(A) divalproex ER 1,027.8 ± 404 mg/d (70.3 ± 27.5 ng/dl)	6 w	—MADRS/MRS: (A):3(33.3%)	(A)7(63.6%)	(A):6(66.7%)
		16(9/7)	(B) 43.3 ± 4.1	(B) 2:6	—MADRS: (A) 25.1 ± 8.5 (B) 29.5 ± 7.6	(B) placebo		(B):1(14.3%)	(B)5(55.6%)	(B):3(42.9%)
Herranz et al., 2006	RCT/crossover design	48(48/48/48/48)	5-14(9 ± 3)	29:19	—EP(with a confident clinical and electroencephalographic diagnosis of)	(A1) Depakine Crono, ChVbid, 873 ± 241 md/d	16 m	—Seizure Frequency (Complete seizure freedom): (A1) :40 (83%)	(A1) :41 (85%)	(A1):18(38)
						(A2) Depakine Crono, ChVom, 871 ± 243 mg/d		(A2) :37 (77%)	(A2) :39 (81%)	(A2):12(25)
						(A3) Depakine Crono, ChVoe 867 ± 245 mg/d		(A3) :36 (75%)	(A3) :38 (79%)	(A3):22(46)
						(B) Depakine, CVbid, twice daily, 874 ± 248		(B): 35 (73%)	(B): 40 (83.3%)	(B):17(35)
Hirschfeld et al., 2010	RCT/placebo controlled	225(147/78)	(A)38.5 ± 11.38	67:80	—BD(DSM-IV)	(A) divalproex ER, 2,210.5 ± 769.48 md/d (77.9 μg/ml)	3 w	—MRS (reduction≥ 50% from the last day of washout period): (A)49(50.3%)	(A): 74(50.3%)	(A):109(74%)
		222(144/78)	(B)40.4 ± 11.28	44:34		(B) placebo		(B)36(46.2%)	(B): 36(46.2%)	(B):54(69%)
Jensen et al., 1994	RCT/placebo controlled	43(22/21)	27–62(46)	37:6	—MA(IHS)	(A) Deprakine Retard, 386.6 μmol/L (64.2 mg/l)	12 w	M—migraine HA rate(reduction of the migraine days to 50%) (A): 22(65%)	Unclear	(A):14(33%)
		34(18:16)				(B) placebo		(B): 7(21%)		(B):7(16%)
Kernitsky et al., 2005	RCT/crossover design	16(16/16)	6–17(11.6 ± 3.3)	6:10	—EP(stable epilepsy)	(A) VPA-ER 1,234 ± 322 mg/d	4 w	—Seizure control rate: (A): 14(89%)	Unclear	Unclear
						(B) Depakote delayed- release tablets or Depakote Sprinkle, 625–4,500 mg/ daily		(B): 14(89%)		
McElroy et al., 2010	RCT/placebo controlled	62(31/31)	(A)35.7 ± 11.3	20:11	—BD(DSM-IV-TR)	(A) VPA-ER 2,091 ± 437 mg/d	8 w	—YMRS (decreased by ≥50%): (A) 14(47)	(A): 13(43.3%)	Unclear
		60(30/30)	(B)37.1 ± 14.6	16:15	—YMRS: (A) 15.9 ± 3.2 (B) 15.0 ± 3.4	(B) placebo		(B) 11(37)	(B): 15(50.0%)	
Muzina et al., 2011	RCT/placebo controlled	54(26/28)	(A)39.2 ± 12.5	11:15	—BD(DSM-IV)	(A) VPA-ER 1606 ± 44 mg/d	6 w	—MADRS (as a 50% decrease in baseline rating on the MADRS): (A)10(38.5%)	(A): 13(50.0%)	Unclear
		54(26/28)	(A)38.8 ± 14.4	12:16	—MADRS: (A) 29.0 ± 5.1 (B) 28.7 ± 4.8	(A) placebo		(B)3(10.7%)	(B): 13(46.4%)	
Sommerville et al., 2003	RCT/crossover design	77(73/74)	18–73(39 ± 10.8)	39:37	—EP(clinical diagnosis)	(A) Depakote ER 2,188 mg/d	11 d	Not state	Not state	(A):10(13.7%)
						(B) Depakote 1,893 mg/d				(B):15(20.3%)
Thibault et al., 2002	RCT/crossover design	44(43/43)	42–65(35.8 ± 9.7)	19:25	—EP(clinical diagnosis)	(A) Depakote ER 1,000–1500 mg/d	6 m	—Seizure Frequency (Seizure free): (A): 40(93%)	Not state	(A):12(27%)
						(B) Depakote		(B):41(95.3%)		(B):8(18%)
Wagner et al., 2009	RCT/placebo controlled	151(77/74)	(A)12.9 ± 2.28	30:44	—BD(DSM-IV-TR)	(A) VPA-ER 1,286 mg/d (27.1 mg/kg)	1 m	—YMRS (defined as a reduction of ≥50% in YMRS scores from baseline to final evaluation**)**: (A)18(24%)	(A): 56(50.0%/)	(A):51(73.7%)/
		144(74/70)	(A)12.8.2 ± 2.2	27:43	—YMRS: (A) 31.0 ± 5.42 (B) 31.3 ± 5.44	(A) placebo		(B)16(23%)	(B): 61(46.4%)	(B):44(82.4%)

EP, epilepsy; BD, bipolar disorder; MA, migraine headache; RCT, randomized controlled trial; VPA-ER, divalproex extended-release; VPA-DR, divalproex delayed-release; ChVbid, chrono VPA-ER twice daily; ChVom, chrono VPA-ER once daily in the morning; ChVoe, chrono VPA-ER once daily in the evening; IHS, International Headache Society guidelines; HA, headache; MRS, The Mania Rating Scale; MSS, Manic Syndrome Scale; BIS, Behavior and Ideation Scale; YMRS, Young Mania Rating scale; MADRS, Montgomery-Asberg Depression Rating Scale; DSS, Depressive Syndrome Scale; GAS, the Global Assessment Scale; m, month; y, year; w, week; DSM, diagnostic and statistical manual of mental disorders.

### 3.2 Quality Assessment of Included Studies

**FIGURE 2:**
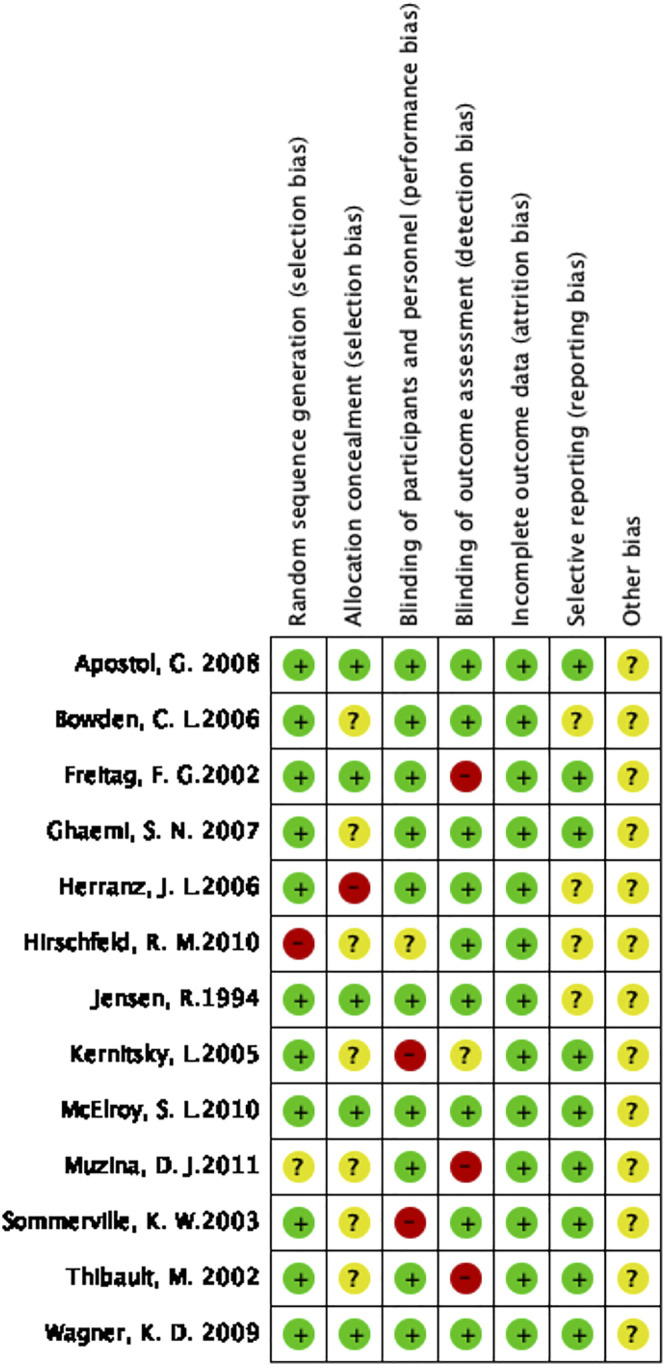
Risk of bias summary.

The quality of the 13 included RCTs was assessed and is depicted in Figure 2.

### 3.3 Pooled Estimates for Outcomes and Subgroup Analysis

#### 3.3.1 Efficacy Assessment

**FIGURE 3:**
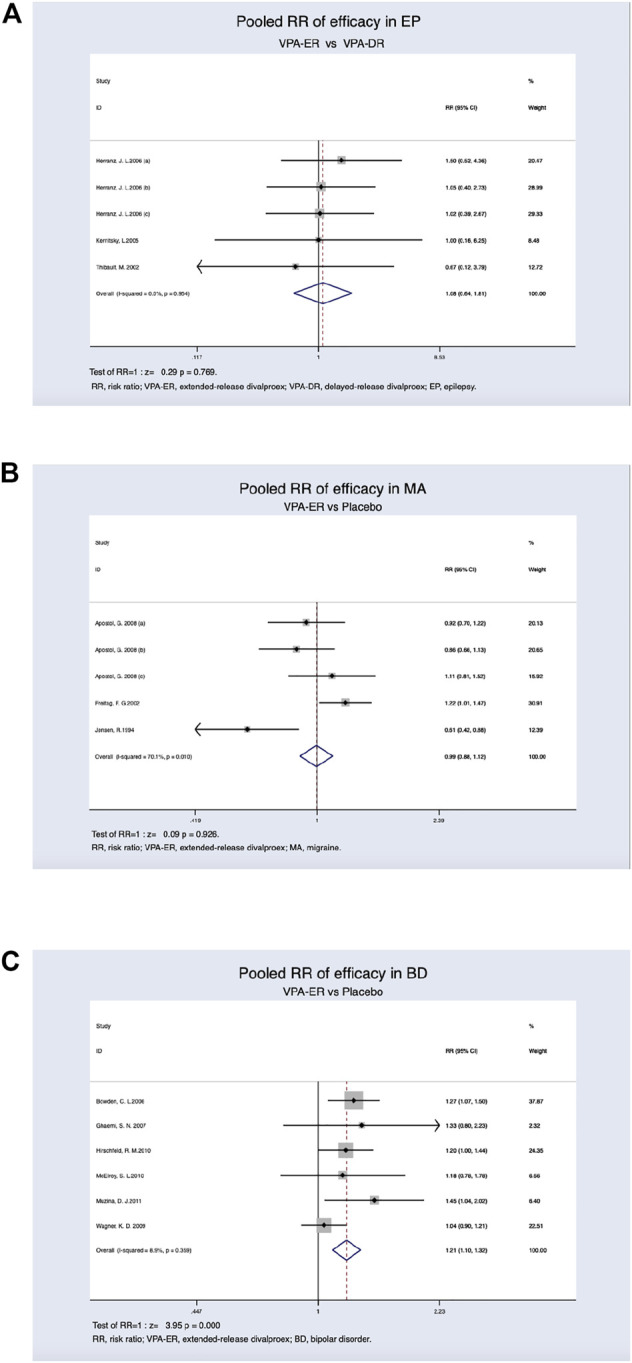
**(A)** Pooled RR of efficacy between VPA-ER with VPA-DR in EP. **(B)** Pooled RR of efficacy between VPA-ER with placebo in MA. **(C)** Pooled RR of efficacy between VPA-ER with placebo in BD.

Therapeutic efficacy was assessed in patients with various diseases. In patients with epilepsy, at least a 50% reduction in the seizure frequency or complete freedom from seizures was achieved. The clinical effectiveness rate in the VPA-ER group was 87.4%, while that of the VPA-DR group was 86.5%. However, the pooled estimated data suggested that patients treated with VPA-ER had no statistical difference compared to those treated with VPA-DA (RR, 1.081; 95% CI, 0.644–1.814; *p* = 0.769) (Figure 3A).

Patients with migraine achieved at least 50% reduction in the migraine headache rate, and this was deemed to be effective, and the response rates were 39.8% for VPA-ER and 40.2% for placebo. We evaluated the RR of the effective rate of VPA-ER compared with that of placebo, but it was shown that VPA-ER did not have an efficacy larger than that of placebo (RR, 0.994; 95% CI, 0.885–1.118; *p* = 0.926) (Figure 3B).

In patients with BD, at least 50% improvement from baseline in the Mania Rating Scale (MRS) scores or Young Mania Rating Scale (YMRS) scores was deemed to be valid data for analysis. Pooled estimated data suggested that patients treated with VPA-ER exhibited a substantially higher response rate than those treated with placebo (RR, 1.207 95% CI, 1.099–1.325; *p* < 0.001) (Figure 3C), and significantly effective rate in the VPA-ER group was 39.5% and in the placebo group was 27.2%. The results of the pooled analysis are summarized in detail in Table 2.

**TABLE 2:** Pooled estimated data of efficacy.

Outcomes	Control group(CG)	No. articles/No. RCTs	No. participants (CG/EG)	Effect estimate RR (95%CI)	I^2^ value (%)	*p* value (Test of RR)
Efficacy assessment						
EP	VPA-DR	3/5	25/24	1.081 (0.664, 1814)	0.00	0.769
	Placebo	–	–	–	–	–
MA	VPA-DR	–	–	–	–	–
	Placebo	3/5	220/231	0.994 (0.885, 1.118)	70.1	0.926
BD	VPA-DR	–	–	–	–	–
	Placebo	6/6	292/290	1.207 (1.099, 1.325)	8.9	0.000

CG, control group; EG, experimental group; RR, Relative risk; CI, confidence interval; EP, epilepsy; BD, bipolar disorders; MA, migraine headache; VPA-DR, delayed-release divalproex.

#### 3.3.2 Safety Assessment

AEs were reported in 14 RCTs, and three only described them rather than reporting the number or incidence (Kernitsky et al., 2005; McElroy et al., 2010; Muzina et al., 2011); these were therefore excluded from the assessment of the safety analysis.

**FIGURE 4:**
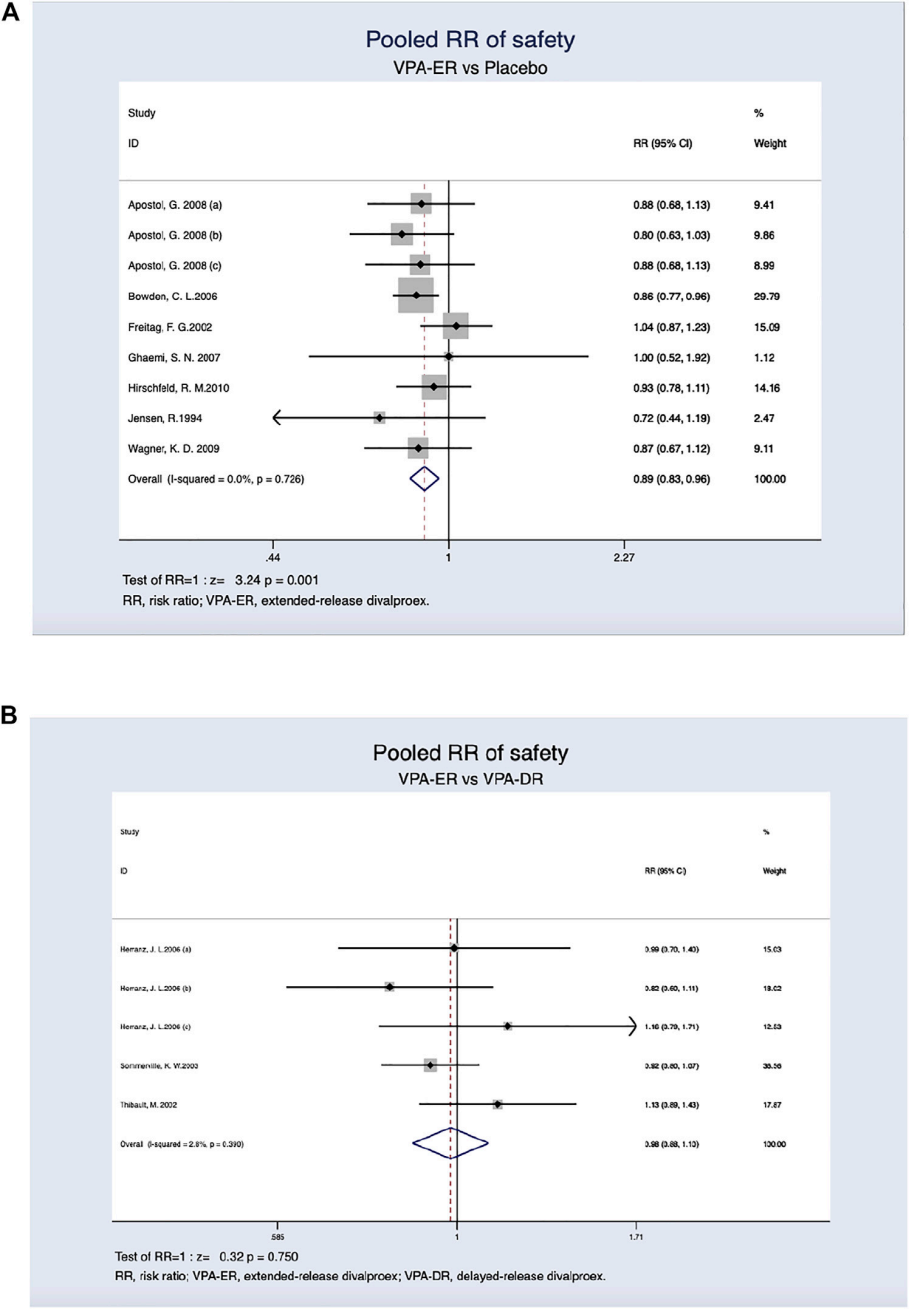
**(A)** Pooled RR of safety between VPA-ER with placebo. **(B)** Pooled RR of safety between VPA-ER with VPA-DR.

Regardless of age and dosage, a lower incidence of AEs was detected in patients treated with the ER formulation (26.8%) than in those treated with placebo (34.8%), and this difference was found to be statistically significant (RR, 0.893; 95% CI, 0.834–0.956; *p* = 0.001) (Figure 4A). Adverse reaction rates were similar in patients receiving VPA-ER and VPA-DR formulations (29.4% *versus* 30.5%). However, the differences were not statistically significant (RR, 0.982; 95% CI, 0.877–1.099; *p* = 0.750) (Figure 4B).

We present separate analyses based on the types of AEs; however, most subgroups comprised only one study. The results shows that people treated with VPA-ER were more likely to have somnolence (RR, 2.110; 95% CI, 1.470–3.027; *p* < 0.001), dizziness (RR, 2.380; 95% CI, 1.395–4.061; *p* = 0.001), nausea (RR, 1.686; 95% CI, 1.255–2.267; *p* = 0.001), vomiting (RR, 2.233; 95% CI, 1.384–3.603; *p* = 0.001), abdominal pain (RR, 2.406; 95% CI, 1.292–4.277; *p* = 0.006), and dyspepsia (RR, 2.099; 95% CI, 1.432–3.075; *p* < 0.001) but were less likely to have constipation (RR, 0.360; 95% CI, 0.156–0.828; *p* = 0.016) (Table 3).

**TABLE 3:** Adverse events (VPA-ER vs. placebo).

System	Adverse events	No. articles/No. RCTs	No. participants (CG/EG)	VPA-ER	Placebo	Effect estimate RR (95%CI)	*p* value (Test of RR)
CNS	Somnolence	7/9	563/808	13.49%	6.39%	2.110 (1.470, 3.027)	<0.001
	Fatigue/Asthenia	5/7	254/419	6.21%	8.66%	0.716 (0.415, 1.237)	0.231
	Headache	5/5	307/456	18.42%	18.13%	1.016 (0.761, 1.356)	0.915
	Dizziness	3/3	236/245	17.14%	7.20%	2.380 (1.395, 4.061)	0.001
	Drowsiness	1/1	41/43	11.63%	4.65%	2.500 (0.513, 12.191)	0.257
	Sedation	3/3	130/149	11.41%	11.56%	0.987 (0.524, 1.857)	0.967
Digestive	Nausea	8/10	556/730	16.99%	10.07%	1.686 (1.255, 2.267)	0.001
	Vomiting	6/10	477/651	9.83%	4.40%	2.233 (1.384, 3.603)	0.001
	Weight increased	5/7	335/502	5.18%	2.99%	1.735 (0.848, 3.551)	0.131
	Appetite increase	3/5	101/99	12.12%	9.90%	1.224 (0.554, 2.703)	0.617
	Abdominal pain	7/9	433/720	6.67%	2.77%	2.406 (1.292, 4.477)	0.006
	Dyspepsia	7/7	533/620	13.39%	6.38%	2.099 (1.432, 3.075)	<0.001
	Diarrhea	7/7	444/560	10.89%	7.88%	1.382 (0.930, 2.054)	0.110
	Dry mouth	4/6	109/109	8.26%	4.59%	1.800 (0.623, 5.198)	0.277
	Constipation	3/5	108/225	4.00%	11.11%	0.360 (0.156, 0.828)	0.016
	Nasopharyngitis/Sinusitis	5/7	477/651	6.45%	5.03%	1.282 (0.788, 2.088)	0.317
Others	Upper respiratory tract infection	3/5	177/337	10.68%	3.95%	2.701 (1.227, 5.945)	0.014
	Influenza	2/4	188/353	4.25%	7.98%	0.533 (0.266, 1.065)	0.075
	Pain/Joint pain/Back pain/Neck pain	4/4	145/364	7.42%	8.57%	0.865 (0.501, 1.495)	0.604
	Rash	1/1	100/62	6.45%	1.00%	6.452 (0.738, 56.408)	0.092

VPA-ER, extended-release divalproex; VPA-DR, delayed-release divalproex; CG,control group; EG, experimental group; RR, Relative risk; CI, confidence interval; CNS, central nervous system.

In addition, when VPA-ER was compared with VPA-DR, the incidences of fatigue/asthenia, headache, memory loss, tremor, weight increase, and hair alterations were lower in the VPA-ER group, and those of nausea, vomiting, appetite increase, constipation, hepatic transaminases increased, and pain were higher, although the differences were not statistically significant (Table 4).

**TABLE 4:** Adverse events (VPA-ER vs. VPA-DR).

System	Adverse events	No. articles/No. RCTs	No. participants (CG/EG)	VPA-ER	VPA-DR	Effect estimate RR (95%CI)	*p* value (Test of RR)
CNS	Fatigue/Asthenia	1/3	48/144	2.78%	6.25%	0.444 (0.103, 1.915)	0.277
	Headache	2/4	122/218	2.29%	3.28%	0.700 (0.191, 2.557)	0.589
	Drowsiness	1/1	48/48	2.08%	2.08%	1.000 (0.093, 10.754)	1.000
	Memory loss	1/1	48/48	0.00%	2.08%	0.333 (0.014, 7.984)	0.498
	Tremor	1/1	48/48	0.00%	2.08%	0.333 (0.014, 7.984)	0.498
Digestive	Nausea	1/1	48/48	2.08%	0.00%	3.000 (0.125, 71.854)	0.498
	Vomiting	1/1	48/48	2.08%	0.00%	3.000 (0.125, 71.854)	0.498
	Weight increased	1/3	48/144	4.86%	8.33%	0.583 (0.178, 1.906)	0.372
	Appetite increase	1/3	48/144	12.50%	8.33%	1.500 (0.534, 4.214)	0.442
	Abdominal pain	1/3	48/144	3.47%	4.17%	0.833 (0.167, 4.156)	0.824
	Constipation	1/1	48/48	2.08%	0.00%	3.000 (0.125, 71.854)	0.498
	Hepatic transaminases increase	1/3	48/144	2.78%	0.00%	3.041 (0.167, 55.481)	0.453
Others	Hair alterations	1/3	48/144	6.94%	8.33%	0.833 (0.274, 2.535)	0.748
	Pain/Joint pain/Back pain/Neck pain	1/1	48/48	2.08%	0.00%	3.000 (0.125, 71.854)	0.498
	Rash	1/1	44/44	0.00%	9.09%	0.111 (0.006, 2.004)	0.137
	Enuresis	1/3	48/144	6.25%	4.17%	1.500 (0.336, 6.702)	0.596

VPA-ER, extended-release divalproex; VPA-DR, delayed-release divalproex; CG, control group; EG, experimental group; RR, Relative risk; CI, confidence interval; CNS, central nervous system.

#### 3.3.3 Retention Rate Assessment

**FIGURE 5:**
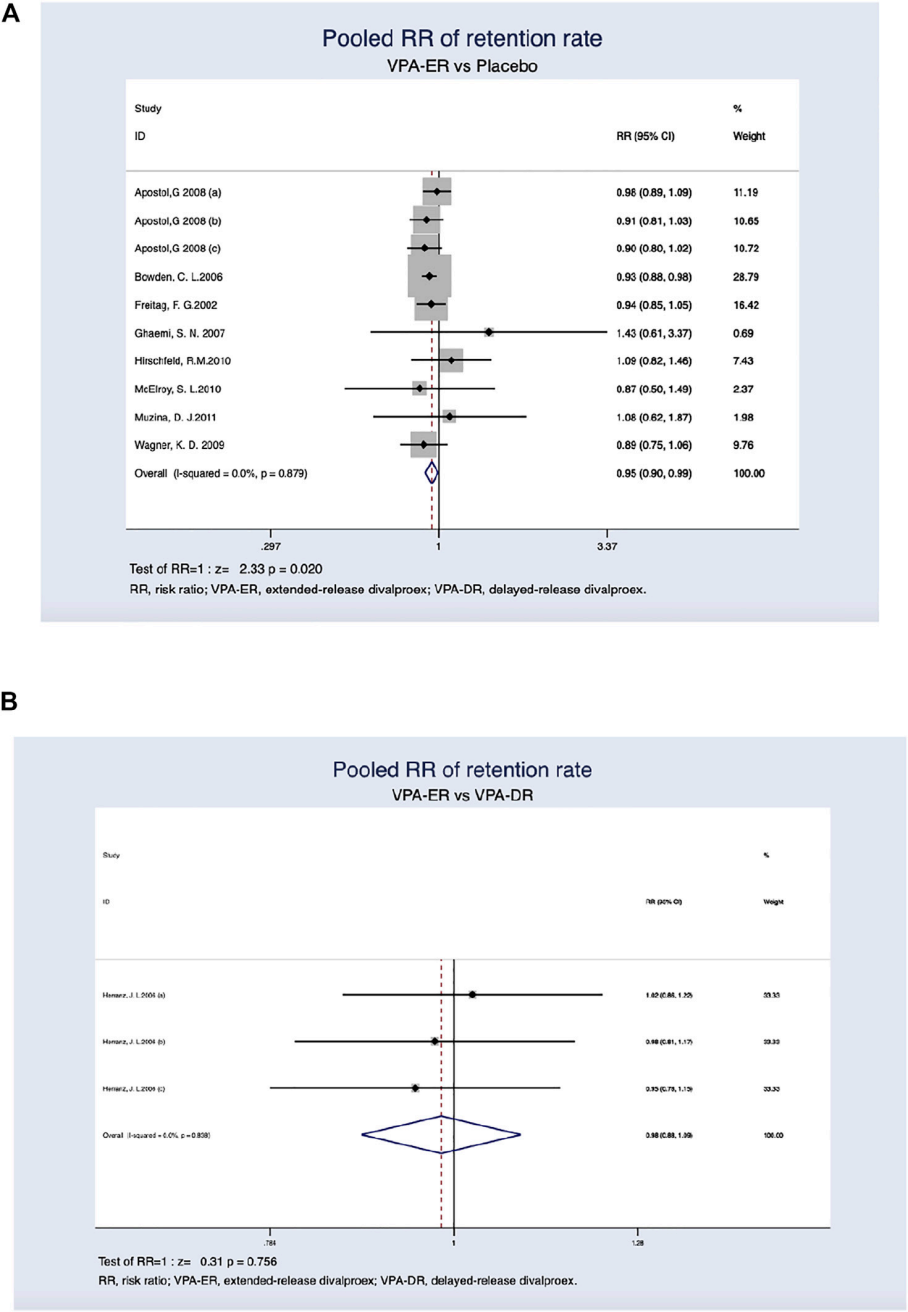
**(A)** Pooled RR of retention rate between VPA-ER with placebo. **(B)** Pooled RR of retention rate between VPA-ER with VPA-DR.

Treatment retention rates in the VPA-ER and placebo groups were 76.0 and 82.7%, respectively. The ratio of all-cause dropout was significantly higher in patients treated with VPA-ER than in those treated with placebo (RR, 0.95; 95% CI, 0.90–0.99; *p* = 0.020) (Figure 5A). The reasons for treatment withdrawal in the VPA-ER group included AEs, non-compliance, suicidality, and others (Table 5).

**TABLE 5:** Reason for discontinuing treatment (VPA-ER vs. placebo).

Reason for discontinuing treatment	No. articles/No. RCTs	No. participants (CG/EG)	VPA-ER	Placebo	Effect estimate RR (95%CI)	*p* value (Test of RR)
Lost to follow up	6/8	329/496	4.23%	4.26%	0.995 (0.513, 1.928)	0.988
Due to adverse event	7/9	514/688	6.98%	4.47%	1.559 (0.961, 2.529)	0.072
Lack of efficacy	8/12	407/558	11.65%	12.04%	0.968 (0.683, 1.370)	0.853
Withdrew consent	4/6	175/344	2.91%	3.43%	0.848 (0.313, 2.295)	0.745
For noncompliance	6/8	398/632	3.32%	2.51%	1.322 (0.629, 2.779)	0.461
Intercurrent illness	1/1	78/147	0.00%	1.28%	0.178 (0.007, 4.317)	0.289
Suicidality	1/1	28/26	7.69%	0.005	5.370 (0.270, 106.877)	0.271
Other reasons	5/7	349/576	6.08%	4.01%	1.515 (0.827, 2.775)	0.179

VPA-ER, extended-release divalproex; CG,control group; EG, experimental group; RR, Relative risk; CI, confidence interval.

In comparison, a single article (Herranz et al., 2006) reported the overall drug retention rates between the VPA-ER and VPA-DR groups. Due to the small sample size and attrition, descriptive analyses were reported. According to a trial (Herranz et al., 2006), both groups had similar drug retention rates (82.0% in VPA-ER versus 83.3% in VPA-DR) (Figure 5B). Lack of efficacy was the main cause of dropout in the VPA-DR group (Table 6).

**TABLE 6:** Reason for discontinuing treatment (VPA-ER.vs.VPA-DR).

Reason for discontinuing treatment	No. articles/No. RCTs	No. participants	VPA-ER	VPA-DR	Effect estimate RR (95%CI)	*p* value (Test of RR)
Lack of efficacy	1/3	48/114	7.64%	6.25%	–	–
Other reasons	1/3	48/114	10.42%	10.42%	–	–

VPA-ER, extended-release divalproex; VPA-DR, delayed-release divalproex; CG, control group; EG, experimental group; RR, Relative risk; CI, confidence interval.

### 3.4 Subgroup Analysis

Despite the absence of statistical heterogeneity, we performed an additional subgroup analysis to determine whether there were significant subgroup differences (Table 7).

**TABLE 7:** Subgroup analysis of outcomes.

Outcomes	Subgroup	No. articles/No. RCTs	No. participants (CG/EG)	Effect estimate RR (95%CI)	I^2^ value (%)	*p* value (Test of RR)
Efficacy assessment						
EP	Total	3/5	25/24	1.081 (0.644,1.814)	0.00	0.769
Duration time	≤4 weeks	1/1	2/2	1.000 (0.160, 6.255)	NA	1.000
	4–12 weeks	1/1	2/3	0.667 (0.117, 3.793)	NA	0.648
	≥12 weeks	1/3	21/19	1.156 (0.654, 2.045)	0.0	0.168
MA	Total	3/5	220/231	0.994 (0.885, 1.118)	70.1	0.926
Duration time	≤4 weeks	1/3	120/131	0.954 (0.810, 1.125)	0.0	0.578
	4–12 weeks	–	–	–	–	–
	≥12 weeks	2/2	100/100	1.047 (0.888, 1.234)	90.9	0.587
BD	Total	6/6	292/290	1.207 (1.099, 1.325)	8.9	0.000
Duration time	≤4 weeks	3/3	239/251	1.187 (1.072, 1.314)	44.4	0.001
	4–12 weeks	3/3	53/39	1.315 (1.039 ,1.664)	0.0	0.022
	≥12 weeks	–	–	–	–	–
Safety assessment						
VPA-DR	Total	3/5	169/174	0.982 (0.877, 1.099)	2.8	0.750
Disease filed	MA	–	–	–	–	–
	BD	–	–	–	–	–
	EP	3/5	169/174	0.982 (0.877, 1.099)	2.8	0.750
Duration time	≤4 weeks	1/1	59/63	0.924 (0.798, 1.070)	NA	0.290
	4–12 weeks	–	–	–	–	–
	≥12 weeks	2/4	110/111	1.015 (0.869, 1.186)	5.3	0.849
Placebo	Total	7/9	451/578	0.893 (0.834, 0.956)	0.0	0.001
Disease filed	MA	3/5	216/251	0.905 (0.812, 1.008)	5.3	0.070
	BD	4/4	235/327	0.882 (0.809, 0.963)	0.0	0.005
	EP	–	–	–	–	–
Duration time	≤4 weeks	4/6	355/475	0.870 (0.806, 0.939)	0.0	0.000
	4–12 weeks	1/1	6/6	1.000 (0.520, 1.922)	NA	1.000
	≥12 weeks	2/2	90/97	0.991 (0.844, 1.164)	44.2	0.916
	≥12 w	2/4	110/111	1.024 (0.876, 1.198)	13.7	0.763
Retention rate assessment						
Placebo	Total	8/10	738/835	0.947 (0.904, 0.991)	0.0	0.020
Disease filed	MA	2/4	334/353	0.936 (0.885, 0.991)	0.0	0.022
	BD	6/6	404/482	0.957 (0.904, 0.991)	0.0	0230
	EP	–	–	–	–	–
Duration time	≤4 weeks	4/6	556/646	0.940 (0.897, 0.990)	0.0	0.018
	4–12 weeks	3/3	67/67	1.027 (0.904, 0.991)	0.0	0.882
	≥12 weeks	1/1	9/11	0.943 (0.848, 1.048)	NA	0.273

VPA-ER, extended-release divalproex; VPA-DR, delayed-release divalproex; CG, control group; EG, experimental group; RR, risk ratio; CI, confidence interval; NA, not applicable; EP, epilepsy patients; BD, bipolar disorders; MA, migraine headache.

We investigated disease efficacy of three different treatment durations, for 4, 4–12, and >12 weeks. The curative efficacy of VPA-ER in treating BD was significantly better than that of the placebo group within 4 weeks (RR, 1.187; 95% CI, 1.072–1.314; *p* = 0.001) and 4–12 weeks (RR, 1.315; 95% CI, 1.039–1.664; *p* = 0.022) during the follow-up period. For migraines and epilepsy, the length of follow-up did not differ between the treatment and control groups.

Safety and AE analyses were subgrouped according to the disease field and duration. The incidence of AEs was significantly lower in patients with BD (RR, 0.882; 95%CI, 0.809–0.963; *p* = 0.005) than in those treated with placebo. However, epilepsy and migraine did not differ significantly between groups. When the duration of therapy was less than 4 weeks, the incidence of side effects was significantly lower in the VPA-ER group (RR, 0.870; 95% CI, 0.806–0.939; *p* < 0.001). Compared with VPA-DR treatment alone, no statistically significant differences were found between the duration points and disease.

For retention rates, subgroup analysis was undertaken according to the treatment duration and disease type. We found significantly lower retention for the VPA-ER groups within 4 weeks of follow-up than the placebo groups (RR, 0.940; 95% CI, 0.897–0.990; *p* = 0.018) and migraine disease (RR, 0.936; 95% CI, 0.885–0.991; *p* = 0.022). In the remaining subgroups, there was no evidence of subgroup differences.

### 3.5 Sensitivity analysis

**FIGURE 6:**
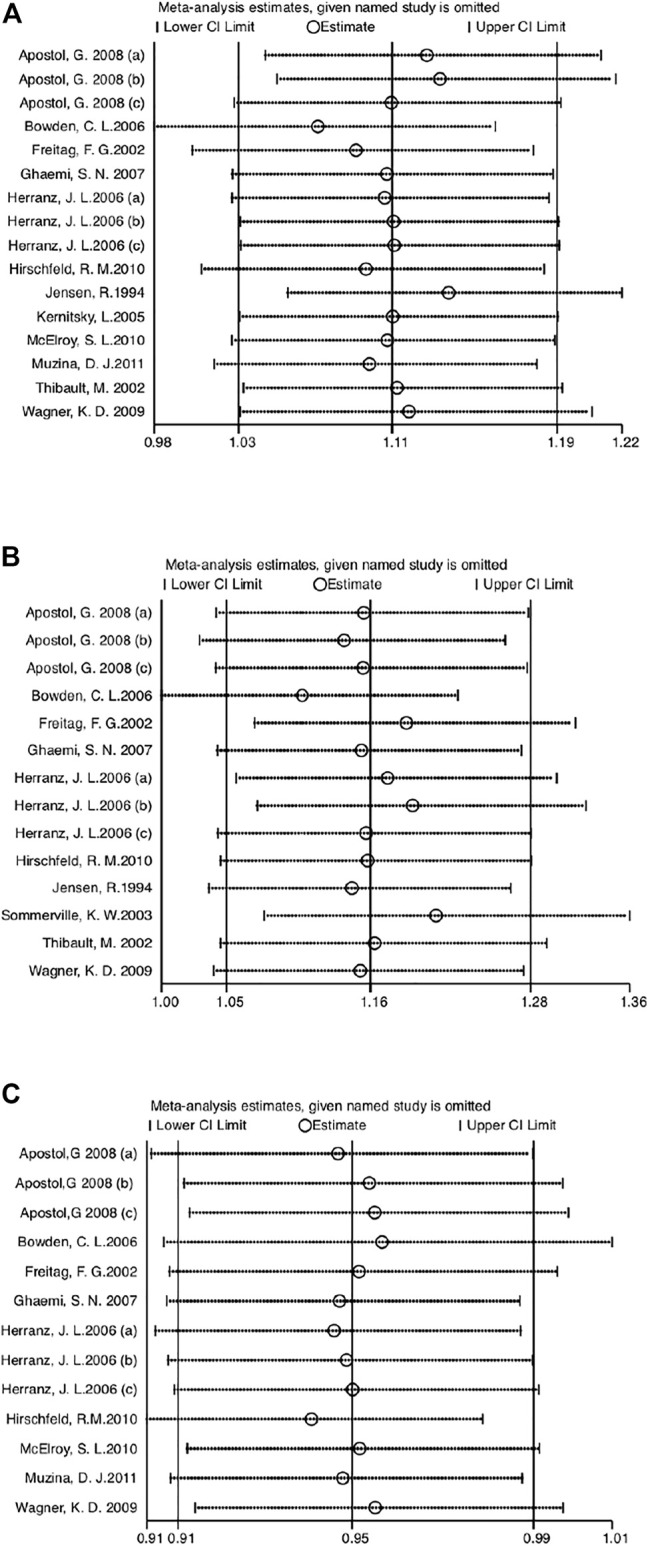
**(A)** Sensitivity analysis of efficacy compared VPA-ER with control groups. **(B)** Sensitivity analysis of safety compared VPA-ER with control groups. **(C)** Sensitivity analysis of retention rate compared VPA-ER with control groups.

Sensitivity analysis was performed by eliminating one study at a time and calculating the pooled estimate of the remaining data. The pooled estimates of efficacy (Figure 6A), incidence of AEs (Figure 6B), and medication compliance (Figure 6C) were not significantly influenced by the elimination method, indicating robust results.

### 3.6 Publication Bias

**FIGURE 7:**
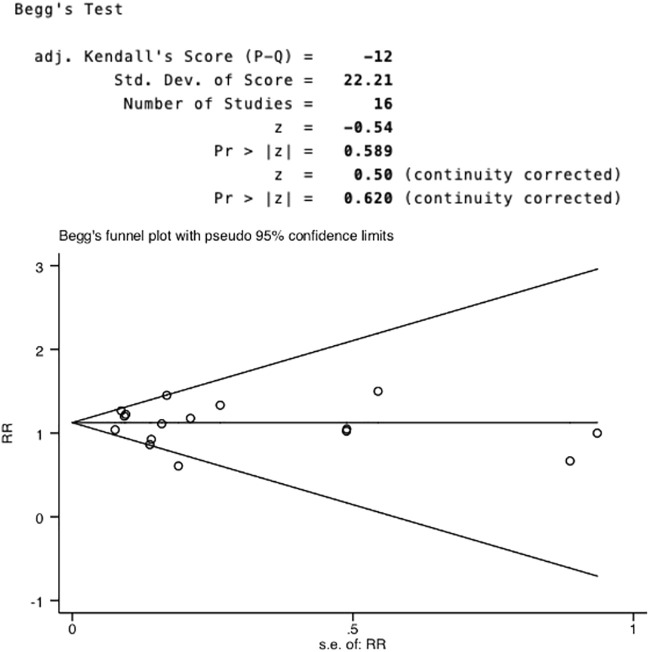
Begg’s funnel plot test of publication bias.

**FIGURE 8:**
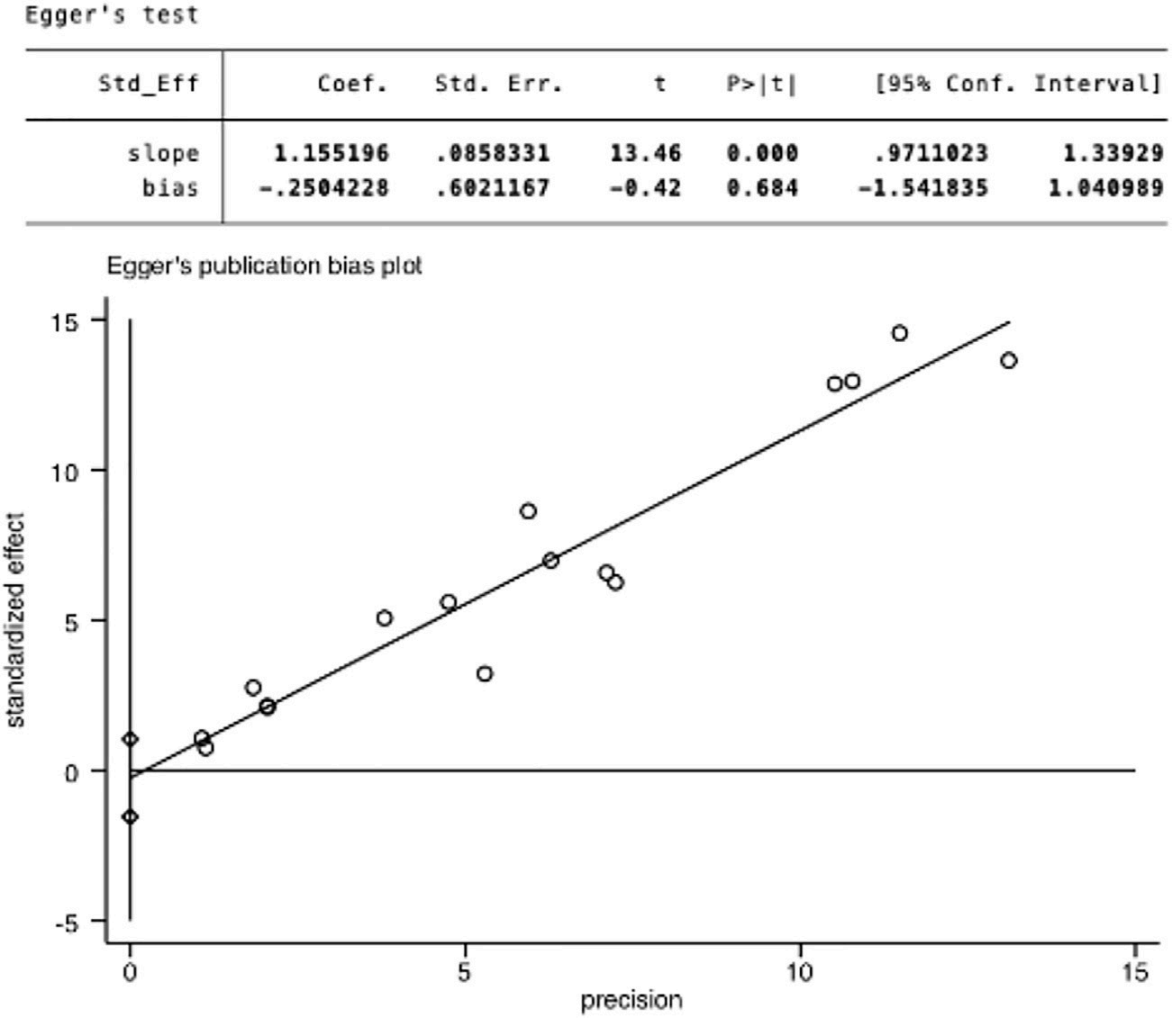
Egger’s linear regression test of publication bias.

Begg’s test (Figure 7) and Egger’s test (Figure 8) were conducted to assess potential publication bias, and no publication bias was examined.

## 4 Discussion

This meta-analysis explored the efficacy, safety, and retention rates of patients treated with VPA-ER *versus* VPA-DR and placebo in 13 RCTs. The pooled analysis demonstrated that VPA-ER could slightly reduce the mean number of migraine days per month compared to placebo. Nevertheless, no significant difference was found in the percentage of patients with ≥50% response rate in headache frequency per month between the VPA-ER and placebo groups. The extended-release (ER) formulation of divalproex sodium has also been approved by the U.S. Food and Drug Administration for migraine prevention in patients >18 years. Several RCTs have revealed that VPA-ER significantly reduced the monthly headache frequency and improved the response rate in adult patients (Klapper, 1997; Freitag, 2003; Kaniecki, 2008; Linde et al., 2013). Jia et al. (2021) also made this conclusion, corroborating the results of our research.

Pooled estimates suggested that VPA-ER had a favorable efficacy, which reduced the baseline values of the rate of MRS scores or YMRS scores in BD patients by at least 50% compared to those in the placebo group (Klapper, 1997; Freitag, 2003; Kaniecki, 2008; Linde et al., 2013; Jia et al., 2021). This is in agreement with the study of Bond et al., which also suggested that divalproex is efficacious in the treatment of BD (Dutta and Zhang, 2004). In addition, VPA can prevent new episodes of BD in adults, as reported by Nestsiarovich et al. (2022). Valproate (VPA) has been used to treat epilepsy and bipolar disorder for many years. Our results also provide evidence that supports VPA-ER had a good efficacy for BD patients.

In the evaluation of clinical efficacy in patients with epilepsy, it has been suggested that VPA-ER treatment confers a higher risk of breakthrough owing to its lower bioavailability (Bialer, 1992; Dutta and Zhang, 2004; Verrotti et al., 2007). However, in our study, we demonstrated that the efficacy of once-daily VPA-ER was superior to that of conventional twice-daily VPA-DR, although the difference was not statistically significant. This is consistent with a previous study showing similar or better efficacy of divalproex ER on seizures and mood disorders in nine open-label trials (Smith et al., 2004).

Reducing the daily dosing frequency can result in a significant increase in treatment compliance (Sommerville et al., 2003). High treatment adherence can significantly improve therapeutic efficacy (Minirth and Neal, 2005). An observational study involving 359 epilepsy patients demonstrated that over 95% of patients administered once-daily evening dosing of valproate sustained-release minitablets had good acceptance (Stefan and Fraunberger, 2005). However, we draw the opposite conclusions from this study, which revealed a significantly inferior drug retention rate in patients treated with VPA-ER to those treated with VPA-DR and placebo, especially in patients with migraine who were treated for less than 4 weeks. While the VPA-ER did not differ with the VPA-DR with regard to the retention rate, and the value was slightly lower than that of VPA-DA, this result was contrary to what we would expect. We performed a detailed analysis of withdrawals and found that the main reasons for dropping out of VPA-ER and placebo included AEs, non-compliance, suicidality, or others and lack of efficacy for VPA-DR. According to Redden et al. (2011), divalproex sodium does not appear to increase the risk of suicide-related AEs, relative to the placebo. However, the U.S. FDA reported that all antiepileptic drugs were related to increased risks of suicidal ideation and behaviors, and clinicians should nonetheless remain vigilant in assessing suicidality (Klein et al., 2021). Overall, the retention rate of VPA-ER was considered acceptable.

In addition, the rate of adverse reactions in the VPA-ER group, especially in the treatment of migraine, was considerably lower than that in the placebo group. Somnolence, dizziness, nausea, vomiting, dyspepsia, and abdominal pain were the primary adverse effects of the VPA-ER treatment, although most AEs were mild or moderate and did not affect the treatment. In addition, we compared the AEs between the VPA-ER and VPA-DR groups. It has been reported that extended-release divalproex sodium can reduce the incidence of AEs associated with divalproex sodium (Stoner et al., 2004), and another retrospective study also reported fewer AEs in patients taking the divalproex ER formulation (Minirth and Neal, 2005). Here, we obtained a consistent finding that the incidence of AEs was slightly lower in VPA-ER than in VPA-DR. Patients receiving VPA-ER had fewer central nervous system adverse effects (i.e., fatigue/asthenia, headache, memory loss, and tremor), weight increase, and hair alterations. At present, a large number of pharmacokinetic studies have shown that the significantly decreased peak-trough fluctuation of plasma valproic acid levels and the stable serum concentrations of divalproex contribute to preventing concentration-dependent AEs (Ieiri et al., 1995; Kondo et al., 2002; Smith et al., 2004; Fagiolino et al., 2007), such as central AEs. Nevertheless, the specific reasons for this need to be further explored.

Some limitations of this meta-analysis should be noted. First, only five RCTs reported VPA-DR as a control, and the placebo was set as the control in the remaining RCTs. Second, some cross-design studies were used, which may have resulted in potential biases, although we performed a sensitivity analysis and publication bias assessment. Third, we did not analyze the drug concentration–effect relationship due to limited availability of data. In the future, we will further explore this based on daily doses in a larger sample over a longer period. Moreover, VPA should be compared to other VPA-ER formulations to identify the optimal formulation.

## 5 Conclusion

This study compared the efficacy, safety, and retention rates of VPA-ER and VPA-DR in various populations. We suggest that the daily dose of VPA-ER is superior in terms of therapeutic efficacy and inferior in terms of AEs, especially on BD, to that of placebo, which provides similar or improved efficacy in controlling seizures, without increasing the occurrence of AEs, compared to conventional VPA-DR formulations. Based on these results, the new ER formulation is recommended because of its better efficacy, medicine compliance, and lower dosing frequency. It can also be used as a substitute for conventional VPA-DR. However, due to its low bioavailability, conversion to divalproex ER requires an appropriate dose increase to avoid subtherapeutic-related problems, and further investigations regarding this aspect of VPA-ER are needed.

## Data Availability

The original contributions presented in the study are included in the article/Supplementary Material, further inquiries can be directed to the corresponding author.
