# Supplementary material for: Efficacy, Safety, and Retention Rate of Extended-Release Divalproex Versus Conventional Delayed-Release Divalproex: A Meta-Analysis of Controlled Clinical Trials
Source: Front Pharmacol. 2022 Apr 5;13:811017. doi: 10.3389/fphar.2022.811017 (PMC9037144; doi:10.3389/fphar.2022.811017)
Supplement: Supplementary file 1 [file DataSheet1.zip › Supplement 1-.Search Strategy.docx]

**Supplement 1. Search terms used in each database**

**MEDLINE via PubMed（1983 to Octber,2021）**

|  | Searches | Results |
| --- | --- | --- |
| #1 | "valproic acid"[MeSH Terms] OR "divalpro*"[Title/Abstract] OR "valpro*"[Title/Abstract] OR ("Depak"[All Fields] AND "e"[Title/Abstract]) OR "Convulsofin"[Title/Abstract] OR "dipropyl acetate"[Title/Abstract] OR "semisodium valproate"[Title/Abstract] OR "Vupral"[Title/Abstract] OR "propylisopropylacetic acid"[Title/Abstract] OR "Ergenyl"[Title/Abstract] OR "magnesium valproate"[Title/Abstract] OR "sodium valproate"[Title/Abstract] OR "calcium valproate"[Title/Abstract] | 21,347 |
| #2 | "Extended release"[Title/Abstract] OR "modified release"[Title/Abstract] OR "delayed release"[Title/Abstract] OR "controlled release"[Title/Abstract] OR "sustained release"[Title/Abstract] OR "prolong release"[Title/Abstract] | 44,324 |
| #3 | "therapeutics"[MeSH Terms] OR "therap*"[Title/Abstract] OR "treatment*"[Title/Abstract] | 9,564,563 |
| #4 | #1 AND #2 AND #3 | 224 |

**EMBASE (1982 to Octber,2021)**

|  | Searches | Results |
| --- | --- | --- |
| #1 | 'valpromide'/exp OR 'valproic acid':ti,ab,kw OR divalpro*:ti,ab,kw OR valpro*:ti,ab,kw OR depak?e:ti,ab,kw OR 'dipropyl acetate':ti,ab,kw OR 'semisodium valproate':ti,ab,kw OR vupral:ti,ab,kw OR 'propylisopropylacetic acid':ti,ab,kw OR ergenyl:ti,ab,kw OR 'magnesium valproate':ti,ab,kw OR 'sodium valproate':ti,ab,kw OR 'calcium valproate':ti,ab,kw | 29,461 |
| #2 | 'sustained drug release'/exp OR 'sustained release preparation'/exp OR 'delayed drug release'/exp OR 'delayed release formulation'/exp OR 'extended release':ti,ab,kw OR 'modified release':ti,ab,kw OR 'controlled release':ti,ab,kw OR 'prolong release':ti,ab,kw | 118,971 |
| #3 | 'therapy'/exp OR 'treatment'/exp OR therapeut*:ti,ab,kw OR therap*:ti,ab,kw | 11,394,969 |
| #4 | #1 AND #2 AND #3 | 316 |

**Cochrane central trails (2001 to Octber,2021)**

|  | Searches | Results |
| --- | --- | --- |
| #1 | MeSH descriptor: [Valproic Acid] explode all trees | 938 |
| #2 | (divalpro*):ti,ab,kw OR (Valpro*):ti,ab,kw OR (Depak?e):ti,ab,kw OR (Convulsofin):ti,ab,kw OR (Dipropyl Acetate):ti,ab,kw | 2,803 |
| #3 | (Semisodium Valproate):ti,ab,kw OR (Vupral):ti,ab,kw OR (Magnesium Valproate):ti,ab,kw OR (Sodium Valproate):ti,ab,kw OR (Calcium Valproate):ti,ab,kw | 781 |
| #4 | (Extended release):ti,ab,kw OR (sustained release):ti,ab,kw OR (delayed release):ti,ab,kw OR (Controlled release):ti,ab,kw OR (Prolong release):ti,ab,kw | 35,606 |
| #5 | MeSH descriptor: [Therapeutics] explode all trees | 317,635 |
| #6 | (Therap*):ti,ab,kw OR (Treatment*):ti,ab,kw OR (Therapeut*):ti,ab,kw | 1,091,904 |
| #7 | #1 OR #2 OR #3 | 2,803 |
| #8 | #5 OR #6 | 1,144,444 |
| #9 | #7 AND #8 AND #4 | 132 |
