# Supplementary material for: Efficacy, Safety, and Retention Rate of Extended-Release Divalproex Versus Conventional Delayed-Release Divalproex: A Meta-Analysis of Controlled Clinical Trials
Source: Front Pharmacol. 2022 Apr 5;13:811017. doi: 10.3389/fphar.2022.811017 (PMC9037144; doi:10.3389/fphar.2022.811017)
Supplement: Supplementary file 1 [file DataSheet1.zip › Supplement 2-Reference list of included studies.docx]

***Reference list of included studies:***

**1.** **Apostol, George (2008)**

- Apostol, G., et al., *Divalproex extended-release in adolescent migraine prophylaxis: results of a randomized, double-blind, placebo-controlled study.* Headache, 2008. **48**(7): p. 1012-1025.

DOI:10.1111/j.1526-4610.2008.01081.x

**2.** **Bowden, C. L. (2006)**

- Bowden, C.L., et al., A randomized, placebo-controlled, multicenter study of divalproex sodium extended release in the treatment of acute mania. Journal of clinical psychiatry, 2006. 67(10): p. 1501‐1510.

DOI: 10.4088/jcp.v67n1003

**3.** **Freitag, Frederick G. (2002)**

- Freitag, F.G., et al., A randomized trial of divalproex sodium extended-release tablets in migraine prophylaxis. Neurology, 2002. 58(11): p. 1652-1659.

DOI: 10.1212/wnl.58.11.1652

**4.** **Ghaemi, S. Nassir (2007)**

- Ghaemi, S.N., et al., Divalproex in the treatment of acute bipolar depression: a preliminary double-blind, randomized, placebo-controlled pilot study. The Journal of clinical psychiatry, 2007. 68(12): p. 1840-1844.

DOI: 10.4088/jcp.v68n1203

**5.** **Herranz, J. L. (2006)**

- Herranz, J.L., et al., Conventional and sustained-release valproate in children with newly diagnosed epilepsy: a randomized and crossover study comparing clinical effects, patient preference and pharmacokinetics. European journal of clinical pharmacology, 2006. 62(10): p. 805‐815.

DOI: 10.1007/s00228-006-0175-2

**6.** **Hirschfeld, R. M. (2010)**

- Hirschfeld, R.M., et al., A randomized, placebo-controlled, multicenter study of divalproex sodium extended-release in the acute treatment of mania. Journal of clinical psychiatry, 2010. 71(4): p. 426‐432.

DOI: 10.4088/JCP.08m04960yel

**7.** **Jensen, R. (1994)**

- Jensen, R., T. Brinck, and J. Olesen, Sodium valproate has a prophylactic effect in migraine without aura: A triple-blind, placebo-controlled crossover study. Neurology, 1994. 44(4): p. 647-651.

**8.** **Kernitsky, L. (2005)**

- Kernitsky, L., et al., Extended-release divalproex in child and adolescent outpatients with epilepsy. Epilepsia, 2005. 46(3): p. 440‐443.

DOI: 10.1111/j.0013-9580.2005.39804.x

**9.** **McElroy, Susan L. (2010)**

- McElroy, S.L., et al., Randomized, double-blind, placebo-controlled study of divalproex extended release loading monotherapy in ambulatory bipolar spectrum disorder patients with moderate-to-severe hypomania or mild mania. The Journal of clinical psychiatry, 2010. 71(5): p. 557-565.

DOI: 10.4088/JCP.08m04854yel

**10.** **Muzina, David J. (2011)**

- Muzina, D.J., et al., Acute efficacy of divalproex sodium versus placebo in mood stabilizer-naive bipolar I or II depression: a double-blind, randomized, placebo-controlled trial. The Journal of clinical psychiatry, 2011. 72(6): p. 813-819.

DOI: 10.4088/JCP.09m05570gre

**11.** **Sommerville, K. W. (2003)**

- Sommerville, K.W., et al., Bioavailability of a Divalproex Extended-Release Formulation versus the Conventional Divalproex Formulation in Adult Patients Receiving Enzyme-Inducing Antiepileptic Drugs. Clinical drug investigation, 2003. 23(10): p. 661‐670.

**12.** **Thibault, Manon (2002)**

- Thibault, M., et al., Divalproex extended-release versus the original divalproex tablet: results of a randomized, crossover study of well-controlled epileptic patients with primary generalized seizures. Epilepsy research, 2002. 50(3): p. 243-249.

DOI:10.1016/s0920-1211(02)00048-7

**13.** **Wagner, Karen Dineen (2009)**

- Wagner, K.D., et al., A double-blind, randomized, placebo-controlled trial of divalproex extended-release in the treatment of bipolar disorder in children and adolescents. Journal of the American Academy of Child and Adolescent Psychiatry, 2009. 48(5): p. 519-532.

DOI: 10.1097/CHI.0b013e31819c55ec
